# Study on optimization of inspection mechanism of concrete beam bridge

**DOI:** 10.1371/journal.pone.0256028

**Published:** 2021-08-12

**Authors:** Dan Su, Yisheng Liu, Xintong Li, Zhicheng Cao

**Affiliations:** 1 Department of Construction Management, School of Economics and Management, Beijing Jiaotong University, Beijing, China; 2 China Astronautics Standards Institute, Beijing, China; Al Mansour University College-Baghdad-Iraq, IRAQ

## Abstract

China is shifting from the stage of large-scale bridge construction to the stage of equal emphasis on the construction and maintenance of bridges. The problems of low efficiency and high cost in bridge inspection are becoming more and more prominent, which threaten people’s life safety. In this paper, the deterioration state prediction model of concrete beam bridge under Boosting DT C5.0 was established as the basis, and optimization suggestions were put forward in terms of bridge inspection standards and processes, which aims to perfect the bridge inspection mechanism, realize the fine management of the bridge and prolong the service life of the bridge. Research shows that: first, the bridge inspection standard with a single index should be improved into the bridge inspection standard with multiple indexes, so as to scientifically determine the bridges that need to be inspected and optimize the allocation of maintenance resources. Second, the bridge deterioration state prediction model is used to add a screening mechanism for the bridge in the inspection plan, which can effectively reduce the workload of bridge inspection and enhance the inspection efficiency. Third, the deterioration phenomenon of coexistence between adjacent traffic assets should be fully considered and the linkage inspection mechanism of adjacent traffic assets should be established to improve the effect of bridge inspection.

## Introduction

Bridges serve as critical structures to the functionality of road networks within an infrastructure system. With the continuous improvement of China’s economic strength, infrastructure construction has been developing rapidly. In recent years, China has made important breakthroughs in the field of bridge construction technology, which also highlights the technical deficiencies of Chinese bridges in the operational stage [1]. Due to the lack of bridge maintenance and management technology, the bridge is prone to structural safety risks, which brings huge hidden dangers to people’s life safety [2, 3]. The focus of bridge engineering in China is changing from construction to maintenance [1]. Bridge inspection is the starting point of bridge maintenance. In order to improve the safety and sustainability of bridge service, it is necessary to perfect the bridge inspection mechanism, improve the efficiency and effect of bridge inspection, so as to accurately grasp the current state of the bridge and lay a solid foundation for the formulation of bridge maintenance plan [4]. Existing research ideas for improving the scientificity of bridge inspection can be roughly divided into two categories: namely Intelligent bridge inspection and rationalization of bridge inspection plan.

With the birth and rise of a new round of scientific and technological revolution and industrial change, modern technologies such as "5G" technology, "Internet +" technology, new sensors, robots and artificial intelligence have brought new opportunities to the innovation of bridge engineering [5], and bridge inspection is developing towards automation and intelligence [6]. Karim et al. [7] and Aliyari et al. [8] proposed to replace labor-intensive bridge detection with UAV mobile camera, and also proposed a structural damage pattern recognition algorithm based on cellular automata. Yang et al. [9] Based on virtual simulation of vehicle-bridge interactions, a bridge inspection method based on vehicle behavior is proposed to reduce labor consumption and improve the efficiency of bridge inspection. Xu et al. [10] proposed to integrate UAV technology, artificial intelligence technology and telecommunications technology into the three-dimensional bridge model to realize the automation of bridge inspection and evaluation. Nguyen et al. [11] integrated a camera and eddy current sensor into an adaptable tank-like robot to realize the detection of deep fatigue cracks in steel structures. Many advanced bridge inspection techniques are still in the exploratory stage. It is important to determine its overall feasibility under practical considerations. However, at the current stage, intelligent bridge inspection is not applicable to all bridge types. Concrete bridges account for more than 90% of the total number of bridges in China, and beam bridges account for more than 74% [12, 13]. The concrete beam bridge is the largest bridge structure in China. Because of its structural characteristics, the medium and small bridges are in the majority. The concrete beam bridge, which has been built for a long time, is the main bridge in urgent need of maintenance at present. However, on the one hand, it is technically difficult to realize intelligent detection because there is no reserved detection equipment track and embedded induction device in the construction process. On the other hand, the cost of intelligent bridge detection is high. In addition, concrete beam bridges are relatively simple in structure, the difficulty of small and medium-sized bridge inspection is low, manual inspection can meet its needs, comprehensive technical, economic and other aspects of consideration, the current concrete beam bridge inspection is still based on manual inspection [14].

Through years of bridge inspection, the bridge management department has accumulated a certain amount of bridge inspection data, which provides data support for data mining. In the face of limited bridge inspection resources, many scholars optimize the inspection mechanism of infrastructure by building structural degradation prediction model, and verify that this method can effectively improve the inspection efficiency and reduce the inspection cost through data. Among them, ANN is often used to construct structure deterioration prediction models. Patrick et al. [15] used the ANN to analyze the bridge operation data, forecast the bridge operation state, and then formulated the corresponding inspection plan. Tatari et al. [16] took data of physical and environmental characteristics of culverts as input factors and an ANN model was used to predict the deterioration of culvert. Stoner et al. [17] used both a logistic regression model and an ANN model to predict the deterioration of culvert. Based on the prediction results, they reduced the frequency of on-site inspection, scientifically planned the priority of culvert inspection, and improved the efficiency of culvert inspection. In addition to ANN, stochastic degradation models, such as CBR and Markov models, have been used in bridge management for many years, specifically to optimize inspection and maintenance timing. Ilbeigi et al. [18] established a two-dimensional Markov process model that takes into account the current state of the bridge and the number of years the bridge has been in that state, and predicts the future state of the bridge based on historical data, in order to determine the optimal bridge inspection interval. Morcous et al. [19] compared CBR with the Markov method and proved that CBR has greater potential in predicting future conditions of infrastructure. Atadero et al. [20] explored a different strategy based on bayesian updating to determine the interval between inspections and the type of inspection technique to use for bridges. The above research proves that it is feasible to optimize the bridge inspection mechanism based on the prediction model of bridge deterioration, but the current research is not perfect. In the modeling of bridge deterioration prediction, with the continuous accumulation of data and the continuous development of machine learning algorithms, a wider range of models can be introduced in addition to ANN. In terms of the optimization of bridge inspection mechanism, the existing research focuses on how to upgrade the fixed inspection interval to a more rational and flexible inspection interval and how to schedule the inspection priority scientifically. The optimization of bridge inspection standards and content has not done more discussion.

The objective of this study is to improve the inspection mechanism of concrete beam bridge and improve the efficiency and effect of bridge inspection. Based on the prediction model of concrete beam bridge deterioration, suggestions are put forward from three aspects of bridge inspection standard, inspection process and inspection content. Selecting appropriate input and output variables, the prediction model of concrete beam bridge deterioration is constructed. Using Accuracy, Precision, F-Score and Recall, the Boosting DT C5.0 with better prediction performance and faster running speed was selected from neural network, support vector machine and decision tree to predict the deterioration of concrete beam bridge. On this basis, the optimization suggestions of bridge detection mechanism are put forward through empirical method, including the construction of multi-index detection standards for bridge, the design of selective detection process for bridge, and the establishment of the linkage detection mechanism of adjacent assets. The results of this study will help the bridge management department to better grasp the operation status of concrete beam bridge, effectively use the bridge inspection resources, and save the cost of bridge inspection.

The remainder of this paper is structured as follows: Firstly, the construction process of bridge deterioration prediction model and algorithm comparison process are described. Then, three suggestions on the optimization of the inspection mechanism of concrete beam bridge are put forward, and the feasibility of these suggestions is proved through empirical analysis. Finally, the results are presented, and future works are recommended.

## Construction of bridge deterioration prediction model

Bridge deterioration refers to the damage of bridges under the action of natural environment and human factors, which leads to the deterioration of the durability of bridges and makes their working performance fail to meet the requirements of relevant standards and specifications [21]. The deterioration of the bridge is related to the formulation of the structural inspection plan. Prediction models of bridge deterioration state can be divided into four categories: physical model, deterministic model, random model and artificial intelligence model [22]. In this paper, the input variables and output variables used for the development of the deterioration state prediction model were reasonably selected through literature review, and three machine learning algorithms, namely artificial neural network, support vector machine and decision tree, were used for model operation.

### Variable selection

Bridge deterioration is a complex process and it is difficult to consider all influencing factors in the model construction. When determining the input variables and output variables of the model, the selection principles of validity and availability should be followed. Different bridge materials and structures have different deterioration factors. In this study, comprehensively consider the deterioration reasons of concrete beam bridge and relevant research conclusions [23–28], and the input and output variables of the deterioration state prediction model of concrete beam bridge are determined as shown in Table 1.

**Table 1:** Variable selection of deterioration prediction model for concrete beam bridge.

The types of variables	The variable name	
Output variables	Bridge technical condition rating	Technical condition rating of deck system
		Technical condition rating of superstructure
		Technical condition rating of the substructure
Input variables	Geometric attribute	Structure length
		Structure width
		Gradient
		Number of spans in main unit
	Structure attributes	Deck structure type
		Type of service
		Wearing surface/protective system
	Operating conditions	Average daily traffic
		Average daily truck traffic
		Vehicle load
	Environmental factor	Amount of precipitation
		The number of days when the minimum temperature is less than 0
		Wind speed
		Temperature
		Humidity
		PH value
	Check and maintain history	Historical bridge rating
		Age

Compared with existing studies [29–31], the input variables selected in this study are sufficient and extensive, covering most of the variables used in existing studies. In addition, environmental factors such as temperature, humidity, wind speed and pH value have significant effects on bridge deterioration through causative analysis. In order to improve the accuracy of the prediction model, the influence of environmental factors has been taken into account and supplemented by environmental factors.

### Data preparation

Artificial intelligence prediction model needs to rely on a large amount of data, and the accuracy of prediction is closely related to the quality of data for model development, so stable and reliable data sources should be selected. In general, most of the initial data collected from the data sources have many problems such as missing data, disorderly classification and uneven distribution, so they are not suitable for direct model development. In order to improve the data quality, a series of cleaning operations, such as preprocessing and reclassification, should be carried out for the collected initial data.

#### (1) Data acquisition channel

In order to improve the accuracy of the prediction of bridge deterioration, the data collection channels should be defined to ensure the authenticity and reliability of the data sources and the stable and sufficient data volume. Since the bridge maintenance management department in China has not disclosed the bridge maintenance data, it is hard to use the bridge maintenance data in China to develop the prediction model. In this paper, the NBI of the United States was selected as the main data source for model development. In the NBI bridge database, the bridge technical condition is evaluated on a 9-point scale, with the bridge technical condition decreasing from 9 to 0.

Considering the impact of environmental factors such as different climate and terrain on bridge deterioration and the demand of data sample size, bridge data from 2000 to 2019 in Texas, which is located in the southern part of the United States, were selected as the initial data for model construction. Texas is the second largest state in the United States, with a developed economy, developed transportation network, the largest bridge stock, and a relatively complex geographical environment. Diverse environmental data provide space for further discussion of the impact of environmental factors on bridge deterioration.

The data of the geometric attributes, structural attributes and operating conditions of the bridge in the input variables and the data of the output variables can directly obtain the original data in NBI. Data on environmental factors is available through the National Center for Environmental Information and Climate Prediction Center of the National Oceanic and Atmospheric Administration, NASA Earth Observations, and other websites.

#### (2) Data preprocessing

Data preprocessing is a process of detecting and eliminating errors and inconsistencies from the original data in order to improve the quality of the modeling data.

Selection of bridge materials and structures. The object of this study is the concrete beam bridge, so it is necessary to select the concrete beam bridge from the source database for analysis. The concrete beam bridge in the database mainly includes: concrete slab beam bridge, concrete multi beam bridge, and other (concrete box beam bridge, T beam bridge) three categories.

Bridge type screening. Maintenance measures will change the deterioration state of the bridge. Considering the diversity of maintenance types and materials, as well as the complex impact of maintenance behavior on the status rating of the bridge is difficult to quantify, bridges with renovation and maintenance history are excluded from the modeling process, and only those bridges in service period and in open state are selected.

Vacancy value deleted. Because the NBI bridge database does not force complete entry of all bridge inspection logs, the data has missing values. If a record contains a missing value for any variable, it will be deleted in the analysis. The deletion can be done in both horizontal and vertical directions. The bridge with missing value can be deleted in the horizontal direction, and the corresponding variable can be deleted in vertical direction. The method can be used for any type of statistical analysis and does not require a special calculation method. The limitation is that it will exclude a large number of original samples. After the above data acquisition process and data preprocessing, the original data of 54,432 bridges is left with data of 5,200 bridges. Although a large number of bridge data were removed in this process, compared with other studies using artificial intelligence models (as shown in Table 2), the remaining sample size of this study is considered to be sufficient.

**Table 2:** Number of bridges used in different studies.

Author	Year	Sample size	Reference
Wang G.	2018	25 monitoring points on one bridge over 8 years	[32]
Fang Y.	2019	3185	[33]
Chen Z.	2015	194	[34]
Soetjipto	2017	235	[35]

#### (3) Data reclassification

After data processing, the amount of data for ratings 0 to 3 was too small for the prediction model to accurately predict these ratings. The concrete bridges are re-divided into two different states. The first group is the bridge from grade 0 to grade 6, which means that the condition of the bridge is poor and it needs to be inspected. The second group is the bridge of grade 7 to grade 9, which means that the condition of the bridge is good and there is no need for frequent inspection and maintenance.

Selection evaluation grade 6 as the cause of the boundary value for the following, the manual according to NBI, level 6 is defined as "a state of relatively satisfied, slight degradation" structure components, level 7 is defined as "in good condition, there is some tiny problem", the description of the contrast level 6 and level 7, grade 6 state of degradation. The regrouped data overcome the influence of lack of specific bridge grade data on the output of model results.

### Model building

**Fig 1:**
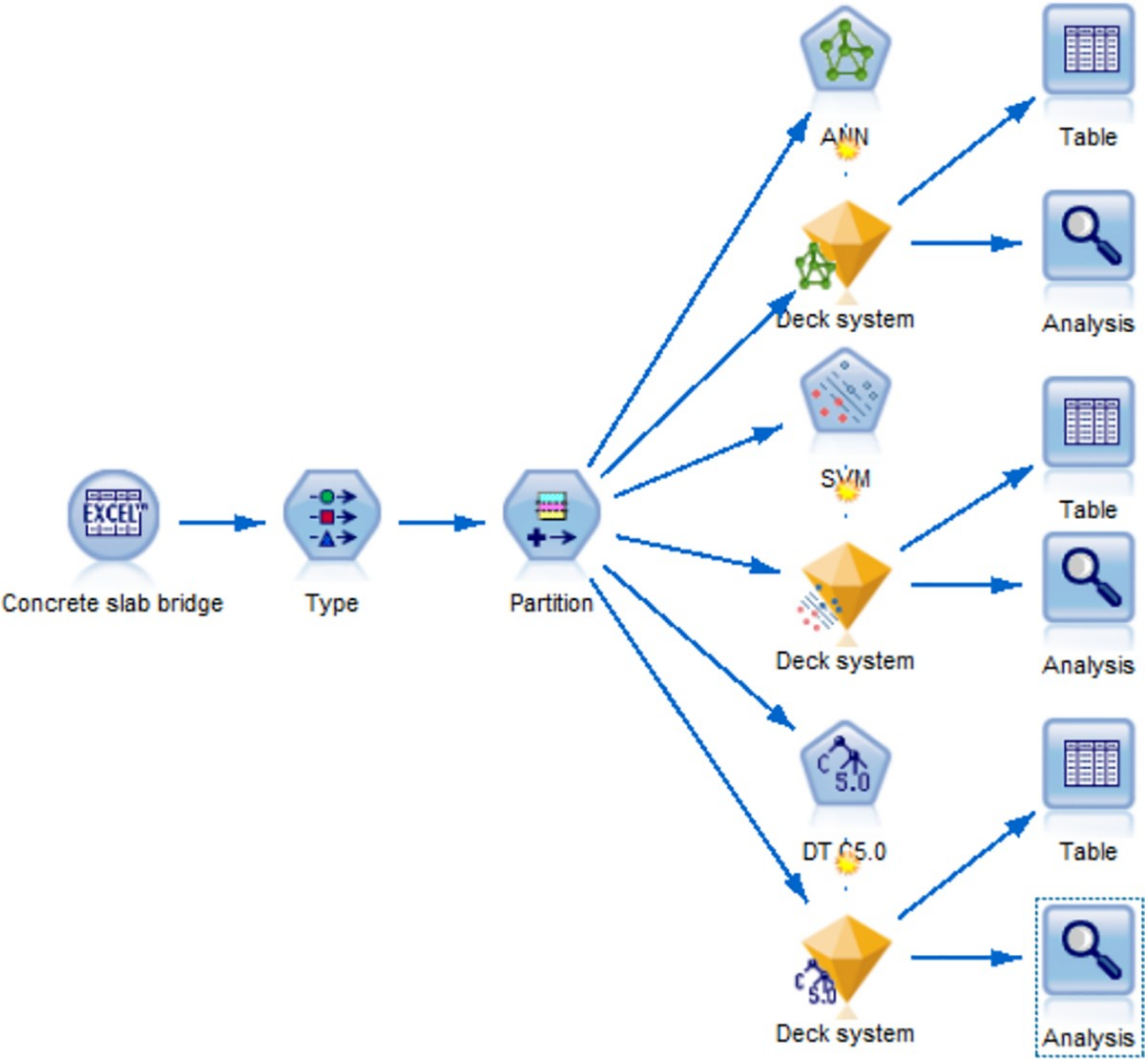
Prediction model of deterioration state of concrete slab bridge.

The corresponding prediction models were established for 3 types of concrete beam bridges and 3 selected output factors of bridges (technical condition of bridge deck system, technical condition of superstructure and technical condition of substructure). The three algorithms of ANN, SVM and DT were used to develop 27 prediction models for the deterioration state of concrete beam bridges (as shown in Fig 1). The model needs to be generalized so that after training it can provide the correct output for data that is not present in the training data or not visible in the model. To avoid overtraining, continuous cross-validation of the model is required.

Step 1: Variable type/role assignment. In this study, SPSS Modeler 18.1, the data mining software of IBM, was used for model development. After the preprocessed data is imported into SPSS Modeler, the first step in the model development process is to assign the types and roles to the variables used (as shown in Table 3).

**Table 3:** Variable types and roles.

The variable name	Variable types	Variable roles
Technical condition rating of deck system	Classification variables	Output variable
Technical condition rating of superstructure	Classification variables	Output variable
Technical condition rating of the substructure	Classification variables	Output variable
Structure length	Numerical variables	Input variable
Structure width	Numerical variables	Input variable
Gradient	Numerical variables	Input variable
Number of spans in main unit	Numerical variables	Input variable
Deck structure type	Classification variables	Input variable
Type of service	Classification variables	Input variable
Wearing surface/protective system	Classification variables	Input variable
Average daily traffic	Numerical variables	Input variable
Average daily truck traffic	Numerical variables	Input variable
Vehicle load	Numerical variables	Input variable
Amount of precipitation	Numerical variables	Input variable
The number of days when the minimum temperature is less than 0	Numerical variables	Input variable
Wind speed	Numerical variables	Input variable
Temperature	Numerical variables	Input variable
Humidity	Numerical variables	Input variable
PH value	Numerical variables	Input variable
Historical bridge rating	Classification variables	Input variable
Age	Numerical variables	Input variable

Step 2: Cross-validation of data. Each model was cross-validated without interruption, and the data was divided into a training set (70% of the data) and a test set (30% of the data) using the "partitioning" function. The training set data was used to create the model, and the test set data was used to test the model.

Step 3: Algorithm parameter adjustment. When building the model, the default settings for each algorithm in the software are used first, and then the parameters are adjusted to improve its predictive performance. Parameters of each algorithm are adjusted as follows:

The ANN algorithm has two important adjustment parameters, that is, the type of activation function and the number of hidden layers. Activation function is the function of the nodes in the neural network to convert input information to output information. In SPSS Modeler, there are two activation functions: MLP and RBF. The MLP with 50 hidden layers was used to build the model and set to continuous training mode until the model’s performance could not be further improved.

An important adjustment parameter of SVM algorithm is the kernel function of the model. There are four kernel functions in SPSS Modeler, including linear function, RBF function, polynomial function and sigmoid function. The kernel function of quadratic polynomial is used in this study.

Boosting DT C5.0 algorithm has two important adjustment parameters, namely, the trimming severity of each subbranch and the minimum number of records. The pruning severity parameter determines the degree to which the decision tree will be pruned. The higher the severity, the simpler the tree. The lower the severity, the more accurate the tree. These two parameters are adjusted according to the output results of each model in the training process.

### Algorithm selection

Selecting appropriate evaluation indexes is the key step of evaluation model prediction performance [36, 37]. Accuracy is often used to evaluate the performance of the prediction model for bridge deterioration [16]. However, the accuracy is not enough to comprehensively evaluate the performance of the prediction model, and it needs to rely on a variety of evaluation indexes to comprehensively evaluate the prediction performance of the model. In this paper, four indexes of Accuracy, Precision, Recall and F-score were selected to evaluate the performance of the prediction model.

### Selection of evaluation indexes

The confusion matrix compares the model predictions with the original data (as shown in Table 4). Where, TN represents the number of negative cases correctly predicted by the model. FN represents the number of positive cases predicted by the model as negative cases. FP represents the number of negative cases predicted by the model as positive cases. TP represents the number of positive cases that the model correctly predicted.

**Table 4:** Confusion matrix.

		Predicted Results	
		Positive	Negative
Actual Data	Positive	TP	FN
	Negative	FP	TN

#### (1) Accuracy

The prediction performance of the prediction model is determined by the number of cases correctly predicted by the model [37]. Its calculation formula is as follows:

$$Accuracy=\frac{TP+TN}{Total}$$ (1.)

In general, higher accuracy means better prediction performance of the model. However, the accuracy also has some limitations, especially when the data sample size is not balanced, the evaluation index provides less information and there are biases. This evaluation bias can be remedied by using additional evaluation indicators.

#### (2) Recall

The recall is the frequency of correct prediction when the case is positive. The low recall rate means that the prediction model generates many FN predictions. Its calculation formula is as follows:

$$Recall=\frac{TP}{TP+FN}$$ (2.)

The recall depends on the number of FN cases, especially when FN cases are the focus of decision makers. The results of recall are particularly important. For example, in the bridge deterioration prediction model, if the positive situation is that the bridge deterioration is serious and needs to be inspected, and the negative situation is that the bridge service condition is good and does not need to be inspected. Then the case of FN is that the model predicts that the bridge is in good service condition, but in fact the bridge deteriorates seriously. At this point, the high number of FN cases means that the model results indicate that many bridges are in good state, when in fact the opposite is true. Such prediction results will mislead decision-makers and make wrong decisions. Moreover, due to the failure to inspect and maintain the seriously deteriorated bridge in time, the potential safety risks of the bridge will be caused and the safety of people’s lives and property will be threatened. The recall can monitor the occurrence of such a situation. The higher the recall rate, the fewer FN cases generated by the model and the higher the reliability of the model.

#### (3) Precision

Precision indicates the correct rate of predicting positive cases; low precision means that the prediction model produces many FP cases. Its calculation formula is as follows:

$$Precision=\frac{TP}{TP+FP}$$ (3.)

The Precision depends on the number of FP cases, especially when FP cases are the focus of decision makers, the results of precision become especially important. Using the same example as the recall rate, the FP case is where the model predicts that the bridge is in a serious state of deterioration, but in fact the bridge is in good condition. At this point, a high number of FP means that the model results show that many bridges are in a state of severe deterioration, which is not the case in fact, and this is a case of low precision. A low accuracy prediction may require additional effort to determine the actual condition of the bridge. Therefore, in order to avoid unnecessary expense, high precision is the guarantee of quality model.

#### (4) F-score

The F-score is a harmonic meaning of Recall and Precision, providing a balanced measure of Recall and Precision. Its calculation formula is as follows:

$$F-Score=2\frac{Precision*Recall}{Precision+Recall}$$ (4.)

The higher the F-score, the less FP and FN predicted by the model, which can correctly predict the actual situation and is not disturbed by false alarm FP. The best value of the F-score is 1, and the worst is 0.

### Evaluation results and analysis

The evaluation results of ANN, SVM and DT algorithms are shown in Table 5.

**Table 5:** Evaluation results of ML prediction model for concrete beam bridge.

	Algorithm	Data Set	Accuracy			Precision			Recall			F-score		
			Ⅰ	Ⅱ	Ⅲ	Ⅰ	Ⅱ	Ⅲ	Ⅰ	Ⅱ	Ⅲ	Ⅰ	Ⅱ	Ⅲ
Deck system	ANN	Train	93%	95%	99%	0.90	0.88	0.99	0.85	0.80	0.99	0.88	0.84	0.99
		Test	88%	91%	91%	0.78	0.76	0.88	0.75	0.68	0.86	0.77	0.72	0.87
	SVM	Train	77%	83%	94%	0.68	0.45	0.92	0.29	0.08	0.91	0.41	0.14	0.92
		Test	77%	82%	90%	0.64	0.44	0.87	0.28	0.09	0.83	0.39	0.15	0.85
	DT	Train	93%	95%	96%	0.90	0.94	0.96	0.87	0.77	0.95	0.88	0.85	0.95
		Test	88%	92%	91%	0.79	0.84	0.91	0.76	0.65	0.86	0.78	0.73	0.89
Super-structure	ANN	Train	96%	91%	99%	0.94	0.84	0.99	0.93	0.80	0.99	0.93	0.82	0.99
		Test	89%	88%	93%	0.84	0.79	0.94	0.82	0.76	0.94	0.83	0.78	0.94
	SVM	Train	76%	57%	93%	0.72	0.34	0.94	0.43	0.81	0.94	0.54	0.48	0.94
		Test	76%	57%	90%	0.71	0.35	0.92	0.43	0.81	0.91	0.53	0.49	0.92
	DT	Train	95%	99%	96%	0.93	0.99	0.96	0.90	0.99	0.98	0.92	0.99	0.97
		Test	90%	95%	91%	0.85	0.92	0.92	0.83	0.87	0.93	0.84	0.89	0.92
Sub-structure	ANN	Train	94%	92%	99%	0.93	0.89	0.99	0.95	0.91	0.99	0.94	0.90	0.99
		Test	88%	89%	91%	0.87	0.85	0.90	0.89	0.87	0.93	0.88	0.86	0.92
	SVM	Train	76%	60%	91%	0.68	-	0.91	0.94	-	0.93	0.79	-	0.92
		Test	76%	60%	87%	0.68	-	0.86	0.93	-	0.91	0.78	-	0.88
	DT	Train	93%	96%	93%	0.92	0.93	0.95	0.92	0.96	0.91	0.92	0.94	0.93
		Test	88%	92%	89%	0.88	0.88	0.91	0.87	0.91	0.87	0.88	0.90	0.89

Note:Ⅰ concrete slab bridge;Ⅱ concrete multi beam bridge;Ⅲ Other concrete beam bridges.

Data source: Calculated according to SPSS Modeler 18.1.

1. In terms of the Accuracy of the models, the overall Accuracy of the 27 models is high and the prediction performance of the models is good, especially the ANN and DT models. The Accuracy of the training set is as high as 92%-99%, and the Accuracy of the test set is also as high as 88%-95%. In contrast, the Accuracy of SVM model is low and unstable, with 57%-94% of the training set and 57%-90% of the test set.
2. From the perspective of the Precision of the model, the overall Precision of the 27 models is high, which means that the deterioration state predicted by the model is in good agreement with the actual state of the bridge. The Precision of the training set of the prediction model developed by ANN and DT algorithm is as high as 0.88–0.99, and the Precision of the test set is also as high as 0.78–0.94. In contrast, the Precision of the prediction model developed by SVM algorithm is generally low and unstable, with the Precision of training set 0.34–0.92 and test set 0.35–0.92.
3. In terms of the Recall of the model, the Recall of the prediction model developed by ANN and DT algorithms is relatively stable, with the mean Recall of the training set at 0.91 and the test set at 0.84. It can be seen that the prediction model developed by ANN and DT algorithms has high reliability. The Recall of the prediction model developed by the SVM algorithm varies greatly among different models. In the prediction model of the deterioration state of the concrete multi beam bridge, the Recall is as low as 0.08 or even impossible to calculate, while in the prediction model of the deterioration state of the other concrete beam bridge, the Recall is as high as 0.93. Since the F-score is calculated according to the recall, the F-score of the prediction model developed by the SVM algorithm is also not ideal, and the F-score of the concrete multi beam bridge is as low as 0.14. The phenomenon that the prediction model has high accuracy and low recall rate is usually caused by the unbalanced data distribution [37].

In conclusion, ANN and DT have better prediction performance and are less affected by data imbalance. Compared with the two, DT has more advantages in terms of computing speed, so Boosting DT C 5.0 is chosen in this study to predict the deterioration state of concrete beam bridge. In existing studies, ANN is often used to predict infrastructure conditions [15–17]. Although DT is rarely used to predict infrastructure conditions, its superior predictive performance has been verified in many fields [38, 39], and has been used and proved to effectively assist the decision-making process of infrastructure [40, 41]. Therefore, it is feasible and meaningful to explore the application of DT in the prediction of bridge deterioration.

## Discussion: Several thoughts on the optimization of inspection mechanism

**Fig 2:**
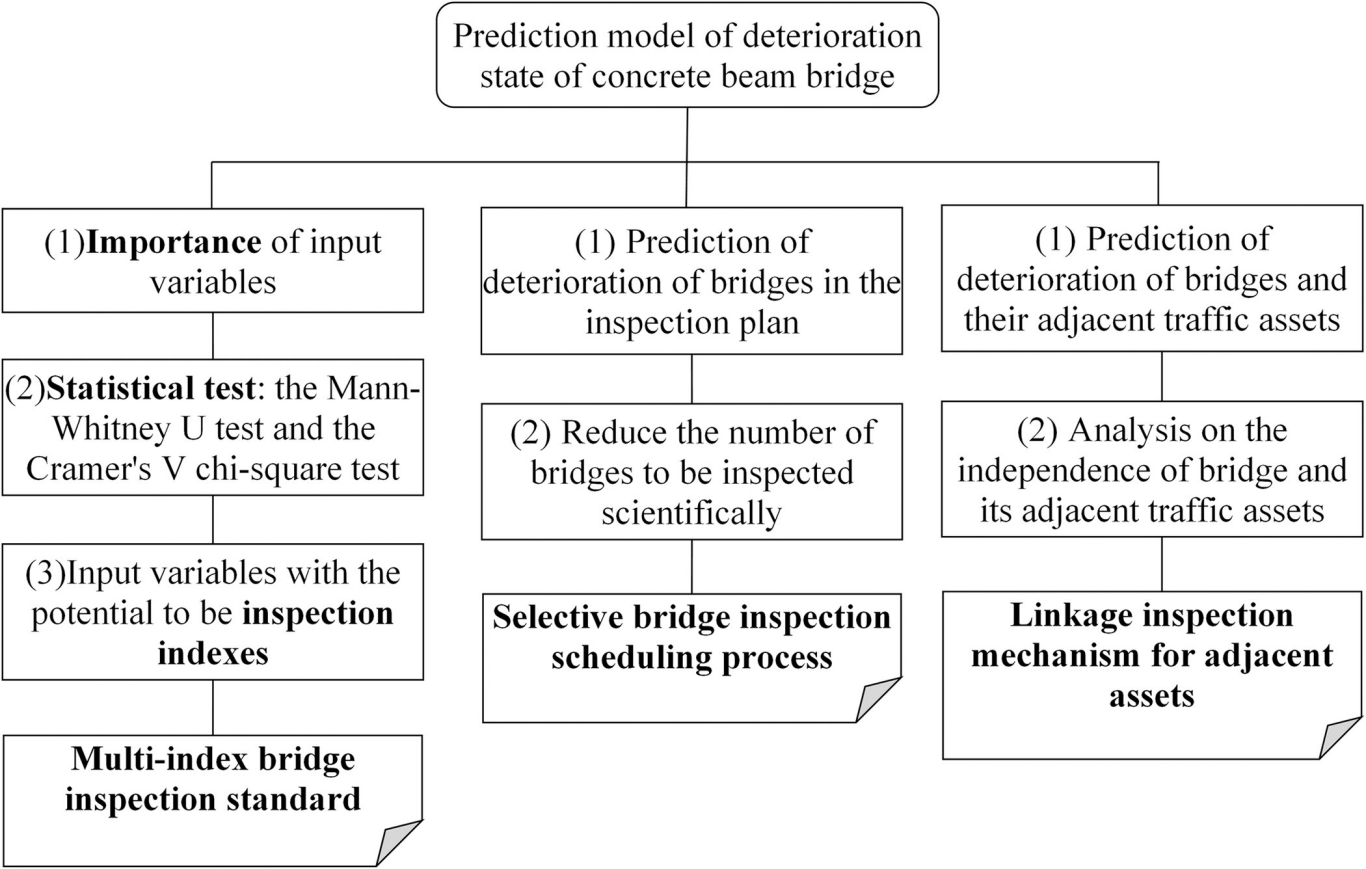
Optimal path of bridge inspection based on prediction of bridge deterioration.

This part discusses the application of the concrete deterioration state prediction model in improving the inspection mechanism of concrete beam bridges (as shown in Fig 2). Suggestions on optimization of bridge inspection are put forward from three aspects: bridge inspection standard, bridge inspection process and bridge inspection content.

### Constructing multi-index bridge inspection standard

*The Code for Maintenance of Highway Bridges and Culverts in China* divides the bridge status into five grades, and stipulates that except for special cases, the inspection interval of bridges is determined by the single index of the status grade of bridges. A regular inspection shall be arranged when the bridge is obviously in grade 3, 4 or 5, and a special inspection shall be arranged when the bridge is in grade 4 or 5, and the longest interval time of inspection shall not exceed three years. The above mentioned regulations adopt the same inspection standard for different bridge characteristics and different environments, which lack pertinence [42].

Based on the deterioration prediction model of concrete beam bridge, other indexes of bridge inspection are excavated to enhance the pertinence of bridge inspection and realize the refinement of bridge inspection. Firstly, the importance of input variables in the degradation prediction model of concrete beam bridge is measured, and the variables with strong importance are selected. Then, according to statistical indicators such as Mann-Whitney U, Chi-square test and Cramer’s V, the distribution of the selected variables in the prediction of concrete beam bridges with different deterioration states can be checked to see if there is a significant difference. The inspection results can provide the basis for the optimization of inspection index of concrete beam bridge.

#### (1) Importance of input variables

The importance of input variables in the DT model (as shown in Table 6). The value range of importance is 0–1, where 0 is the least important and 1 is the most important. According to the *Standard for Evaluating the Technical Status of Highway Bridges*, the weights of bridge deck system, superstructure and substructure are 0.2, 0.4 and 0.4 respectively. Based on this, the comprehensive importance degree of input variables is calculated (the comprehensive importance degree = bridge deck system importance *0.2+ superstructure *0.4+ substructure *0.4).

**Table 6:** Importance of input variables in DT model of concrete beam bridge.

Input variables	Importance												
	The deck system			Superstructure			Substructure			Comprehensive			Mean value
	Ⅰ	Ⅱ	Ⅲ	Ⅰ	Ⅱ	Ⅲ	Ⅰ	Ⅱ	Ⅲ	Ⅰ	Ⅱ	Ⅲ	
Historical bridge rating	0.35	0.34	0.02	0.49	0.06	0.22	0.62	0.81	0.26	0.51	0.42	0.20	0.38
Age	0.20	0.21	0.05	0.08	0.08	0.02	0.06	0.03	0.10	0.10	0.09	0.06	0.08
Average daily traffic	0.08	0.17	0.13	0.04	0.06	0.06	0.01	0.00	0.05	0.04	0.06	0.07	0.06
Gradient	0.03	0.10	0.09	0.07	0.06	0.09	0.04	0.01	0.00	0.05	0.05	0.05	0.05
Structure length	0.06	0.01	0.06	0.03	0.07	0.18	0.03	0.04	0.09	0.04	0.05	0.12	0.07
Structure width	0.07	0.03	0.05	0.01	0.06	0.07	0.02	0.00	0.02	0.03	0.03	0.05	0.04
Number of spans in main unit	0.11	0.04	0.03	0.02	0.05	0.01	0.04	0.02	0.12	0.05	0.04	0.06	0.05
Vehicle load	0.00	0.00	0.00	0.00	0.05	0.00	0.00	0.00	0.01	0.00	0.02	0.00	0.01
Average daily truck traffic	0.01	0.01	0.01	0.01	0.05	0.02	0.02	0.00	0.02	0.01	0.02	0.02	0.02
Deck structure type	0.00	0.00	0.01	0.00	0.00	0.02	0.00	0.00	0.01	0.00	0.00	0.01	0.00
Type of service	0.00	0.00	0.13	0.04	0.05	0.09	0.07	0.00	0.00	0.04	0.02	0.06	0.04
Wearing surface/protective system	0.01	0.00	0.01	0.02	0.05	0.02	0.00	0.00	0.01	0.01	0.02	0.01	0.01
Temperature	0.00	0.01	0.07	0.02	0.07	0.00	0.01	0.03	0.06	0.01	0.04	0.04	0.03
Humidity	0.02	0.00	0.08	0.06	0.05	0.04	0.02	0.01	0.07	0.04	0.02	0.06	0.04
Amount of precipitation	0.04	0.00	0.13	0.00	0.06	0.04	0.00	0.00	0.01	0.01	0.02	0.05	0.03
Wind speed	0.01	0.01	0.04	0.00	0.06	0.00	0.02	0.02	0.05	0.01	0.03	0.03	0.02
The number of days when the minimum temperature is less than 0	0.00	0.01	0.01	0.06	0.05	0.13	0.01	0.02	0.06	0.03	0.03	0.08	0.05
PH value	0.01	0.02	0.07	0.03	0.06	0.00	0.03	0.00	0.05	0.03	0.03	0.03	0.03

Note:Ⅰ concrete slab bridge;Ⅱ concrete multi beam bridge;Ⅲ Other concrete beam bridges.

Data source: Calculated according to SPSS Modeler 18.1

It is found that although the variables affecting the deterioration state of the bridge are different. However, on the whole, the concrete beam bridge is greatly affected by four variables: historical bridge rating, age, structure length, average daily traffic. Their comprehensive importance was 0.38, 0.08, 0.07, 0.06, respectively. In addition, environmental variables such as humidity and the number of days when the minimum temperature is less than 0 also play an important role.

#### (2) Statistical test results and analysis

Two statistical tests, the Mann-Whitney U test and the Cramer’s V chi-square test, were used in this study, both using a significance level of 0.01. If the test result P value is greater than 0.01, the H_0_ of the test will be accepted; otherwise, the H_0_ will be rejected. SPSS Statistics 26 was used to conduct Mann-Whitney U test on each input variable, and the test results were shown in Table 7. Since the test variable of Mann-Whitney U cannot be classified variable, the three variables in the input variables of deck structure type, type of service and wearing surface/protective system cannot be tested.

**Table 7:** Summary of P values from the Mann‐Whitney U test.

Input variables	Mann-Whitney U tests P values								
	Deck system			Superstructure			Substructure		
	Ⅰ	Ⅱ	Ⅲ	Ⅰ	Ⅱ	Ⅲ	Ⅰ	Ⅱ	Ⅲ
Historical bridge rating	.000	.000	.000	.000	.000	.000	.000	.000	.000
Age	.000	.000	.000	.000	.000	.000	.000	.000	.000
Average daily traffic	.000	.000	.139	.000	.000	.805	.000	.000	.001
Gradient	.000	.000	.001	.000	.000	.362	.000	.000	.001
Structure length	.000	.000	.000	.000	.000	.000	.000	.000	.000
Structure width	.984	.000	.000	.748	.000	.000	.000	.000	.000
Number of spans in main unit	.000	.000	.687	.000	.000	.000	.000	.000	.000
Vehicle load	.010	.598	.064	.025	.683	.063	.002	.917	.022
Average daily truck traffic	.000	.060	.000	.000	.000	.000	.000	.000	.000
Temperature	.000	.000	.011	.000	.000	.000	.000	.000	.000
Humidity	.710	.923	.000	.068	.365	.000	.054	.000	.001
Amount of precipitation	.000	.000	.000	.000	.000	.231	.000	.000	.008
Wind speed	.000	.000	.000	.000	.000	.211	.002	.000	.044
The number of days when the minimum temperature is less than 0	.000	.000	.000	.000	.000	.000	.000	.000	.000
PH value	.000	.000	.150	.000	.000	.580	.000	.000	.051

Note:Ⅰconcrete slab bridge;Ⅱconcrete multi beam bridge;Ⅲ Other concrete beam bridges.

Data source: Calculated according to SPSS Statistics 26.

For the same type of concrete beam bridge, the influence of the same variable on the deterioration state prediction of different parts of the bridge is different. For example, for the concrete multi beam bridge, the influence of humidity on the deterioration of the deck system and superstructure is not significant, but the influence on the substructure is significant. For the concrete slab bridge, the P values of humidity and structure width are not significant, so the original hypothesis should be retained. For the concrete multi beam bridge, the P values of vehicle load and humidity are not significant, so the original hypothesis should be retained. For other concrete beam bridges, the P values of the four variables of average daily traffic, vehicle load, pH value and wind speed are not significant, so the original hypothesis should be retained. This result shows that the same variable has different effects on different types of concrete beam bridges. However, in general, the historical bridge rating, age, average daily traffic, gradient, structure length, number of spans in main unit, average daily trunk traffic, temperature, amount of precipitation, wind speed, the number of days when the minimum temperature is less than 0, pH value are significant, and have the potential to be used as the inspection index of concrete beam bridge.

SPSS Statistics 26 was used to perform Chi-square and Cramer’s V tests on each input variable, and the test results were shown in Table 8.

**Table 8:** Chi-square and Cramer’s V test results.

Input variables	Method	Deck system			Superstructure			Substructure		
		Ⅰ	Ⅱ	Ⅲ	Ⅰ	Ⅱ	Ⅲ	Ⅰ	Ⅱ	Ⅲ
Historical bridge rating	Chi-square	.000	.000	.000	.000	.000	.000	.000	.000	.000
	Cramer’s V	.311	.308	.225	.387	.501	.438	.538	.694	.353
Age	Chi-square	.000	.000	.000	.000	.000	.000	.000	.000	.000
	Cramer’s V	.230	.224	.300	.248	.300	.160	.309	.356	.195
Average daily traffic	Chi-square	-	-	.000	-	-	.000	-	-	.000
	Cramer’s V	-	-	.676	-	-	.705	-	-	.657
Grandient	Chi-square	.000	.000	.000	.000	.000	.000	.000	.000	.000
	Cramer’s V	.141	.066	.373	.162	.086	.318	.178	.091	.262
Structure length	Chi-square	.000	.000	.000	.000	.000	.000	.000	.000	.000
	Cramer’s V	.331	.312	.656	.322	.307	.631	.383	.360	.590
Structure width	Chi-square	.000	.000	.000	.000	.000	.000	.000	.000	.000
	Cramer’s V	.201	.194	.351	.201	.218	.298	.257	.141	.319
Number of spans in main unit	Chi-square	.000	.000	.000	.000	.000	.000	.000	.000	.000
	Cramer’s V	.130	.168	.273	.116	.160	.209	.194	.232	.258
Vehicle load	Chi-square	.083	.645	.064	.004	.501	.063	.010	.344	.022
	Cramer’s V	.014	.005	.027	.020	.006	.027	.018	.007	.033
Average daily truck traffic	Chi-square	.000	.000	.000	.000	.000	.000	.000	.000	.000
	Cramer’s V	.138	.102	.316	.137	.126	.397	.198	.098	.313
Deck structure type	Chi-square	.006	-	.000	.284	-	.000	.038	-	.000
	Cramer’s V	.014	-	.095	.006	-	.095	.011	-	.074
Type of service	Chi-square	.000	.000	.000	.000	.000	.000	.000	.000	.000
	Cramer’s V	.061	.020	.304	.088	.121	.156	.150	.056	.090
Wearing surface/protective system	Chi-square	.000	.000	.000	.000	.000	.004	.000	.000	.003
	Cramer’s V	.044	.024	.062	.061	.070	.042	.034	.027	.043
Temperature	Chi-square	.000	.000	.000	.000	.000	.000	.000	.000	.000
	Cramer’s V	.310	.283	.523	.294	.280	.485	.304	.252	.425
Humidity	Chi-square	.000	.000	.000	.000	.000	.000	.000	.000	.000
	Cramer’s V	.059	.067	.131	.079	.052	.226	.073	.075	.197
Amount of precipitation	Chi-square	.000	.000	.000	.000	.000	.000	.000	.000	.000
	Cramer’s V	.340	.301	.525	.327	.298	.479	.314	.277	.426
Wind speed	Chi-square	.000	.000	.000	.000	.000	.000	.000	.000	.000
	Cramer’s V	.298	.272	.514	.288	.273	.483	.278	.245	.423
The number of days when the minimum temperature is less than 0	Chi-square	.000	.000	.000	.000	.000	.000	.000	.000	.000
	Cramer’s V	.190	.187	.358	.183	.189	.356	.172	.176	.280
PH value	Chi-square	.000	.000	.000	.000	.000	.000	.000	.000	.000
	Cramer’s V	.112	.150	.284	.114	.125	.300	.141	.114	.237

Note:Ⅰ concrete slab bridge;Ⅱ concrete multi beam bridge;Ⅲ Other concrete beam bridges.

Data source: Calculated according to SPSS Statistics 26.

Due to data limitations, average daily traffic and deck system type results cannot be output normally. It can be seen from the results of the chi-square test that there is a strong correlation between other input variables except vehicle load and the deterioration state of concrete beam bridge. Cramer’s V test results show that for the same type of concrete beam bridge, the dependence of the same variable on the deterioration state of different parts of the bridge is not significant. For different types of concrete beam bridges, the dependence degree of each variable is slightly different. However, on the whole, seven variables of historical bridge rating, age, structure length, structure width, temperature, amount of precipitation and wind speed are highly dependent on the deterioration state of concrete beam bridge.

#### (3) Analysis of inspection index of concrete beam bridge

Since the number of concrete slab bridges and concrete multi beam bridges accounts for the vast majority, the analysis results of these two types of bridge are more general, and the analysis results of other concrete beam bridges are only for reference. Based on the comprehensive significance of the variables and the statistical test results, the historical bridge rating, age and structure length not only have higher significance, but also show obvious distribution difference, significant correlation and dependence in the statistical test, so they are suitable for the bridge inspection indexes. In addition, the three environmental characteristic variables of temperature, precipitation and wind speed, although their importance is not significant in the prediction process, have the potential to become bridge inspection indexes from the perspective of statistical tests. It can be seen that in addition to the state grade of the bridge, bridge characteristics such as age and length as well as environmental factors such as temperature, precipitation and wind speed also need to be paid attention to when making the bridge inspection plan. Bridge management departments can classify bridges according to bridge characteristics and environmental indicators, and carry out differentiated inspection according to different types of bridges to enhance the pertinence of bridge inspection.

### Optimize bridge inspection process

Based on the deterioration state prediction of concrete beam bridge, the existing inspection process of concrete beam bridge is improved, and the improved inspection process is simulated with actual data, which shows the potential advantages of selective inspection process.

#### (1) Selective bridge inspection scheduling process

**Fig 3:**
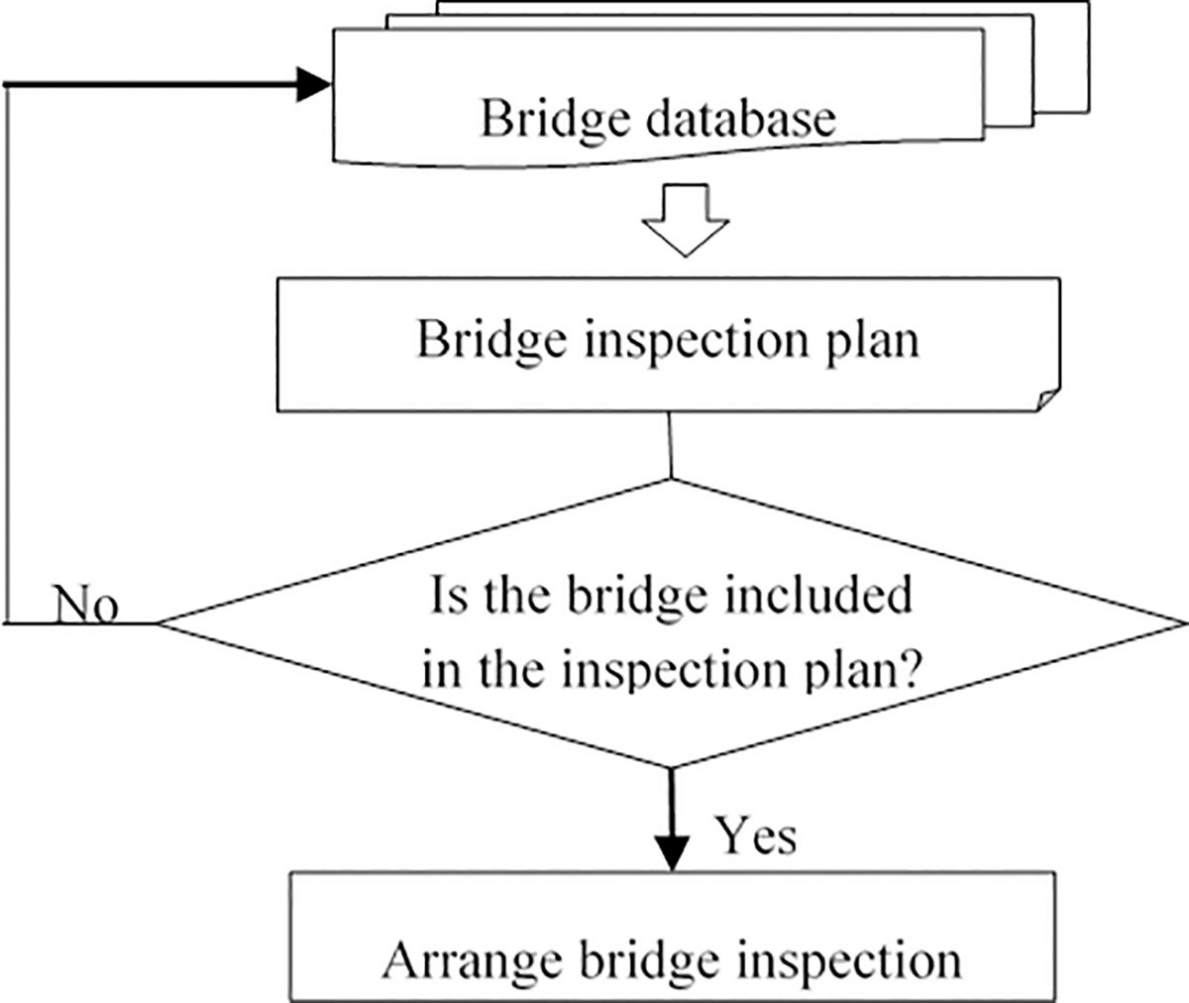
Current highway bridge inspection scheduling process.

According to the current regulations, the inspection and scheduling process of bridges is shown in Fig 3. Running checks are generally not less than once a month. Regular inspection shall be arranged when the defect of important parts (structures) is found to reach the grade 3,4,5 technical condition obviously by running check. The interval between regular inspection shall not exceed three years. Special inspection shall be carried out when the technical condition of the bridge is in grade 4,5 or when it suffers from accidents and other special circumstances. The regular inspection plan of bridges is formulated mainly according to the technical condition of bridges and the prescribed inspection period. The bridges listed in the inspection plan will not undergo secondary screening and will be directly inspected.

**Fig 4:**
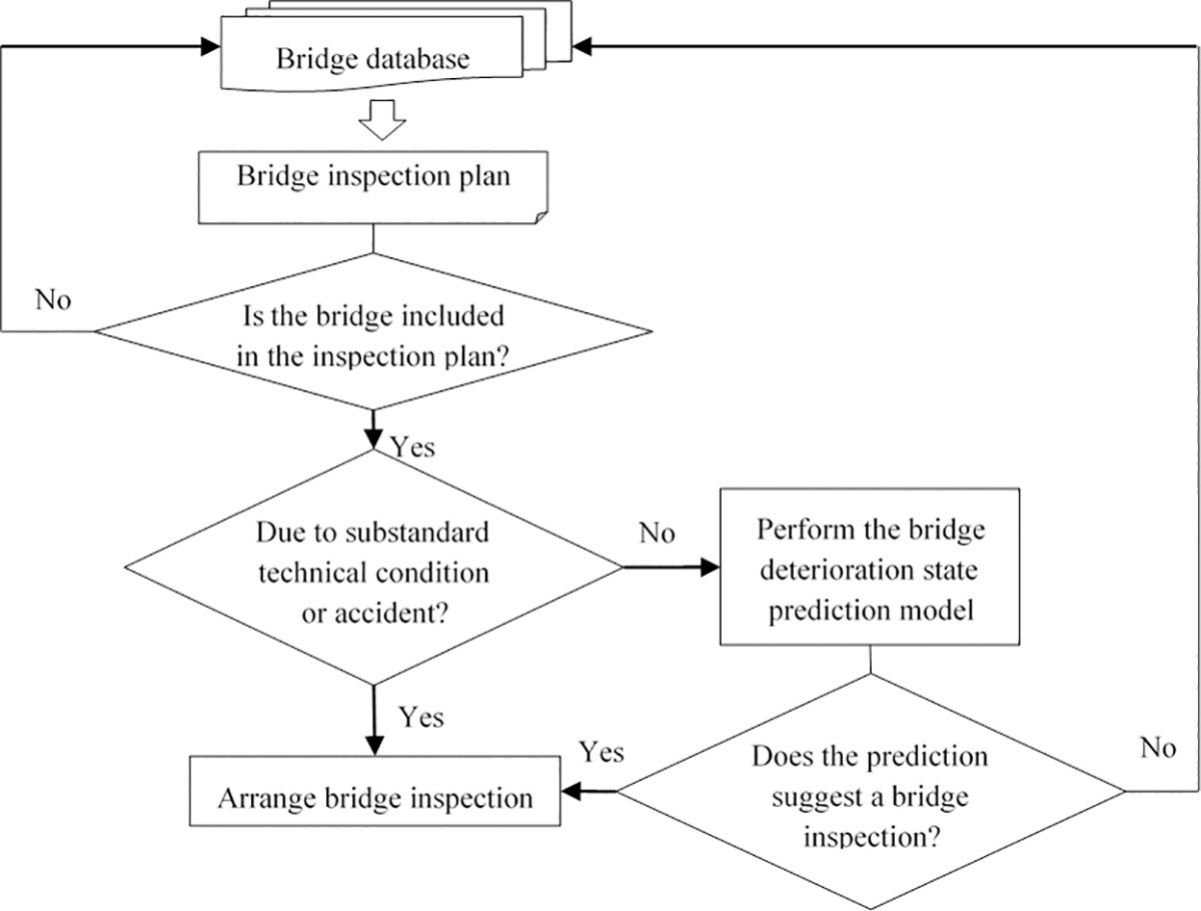
Improved highway bridge inspection scheduling process.

This arrangement has resulted in some bridges in good condition being included in the inspection schedule as they reach their regular inspection deadlines, resulting in a waste of inspection resources. On the basis of the traditional bridge inspection process, this study adds a screening mechanism to the inspection plan. The improved bridge inspection scheduling process proposed in this study is shown in Fig 4.

First of all, the bridges included in the inspection plan are classified, and the bridges included in the inspection plan due to poor technical conditions or accidental accidents such as impact, flood and earthquake are inspected in time, because such bridges have been in a critical state of quality and safety. Secondly, the established bridge deterioration state prediction model is used to deal with the bridges whose technical condition has not reached the critical state but the inspection interval has reached three years, so as to predict their state (0 or 1). Finally, only the bridges with the predicted status of 0 need to be inspected.

#### (2) The empirical analysis

The concrete beam bridges that need to be included in the inspection plan in the next year are determined by the two indexes of "last inspection year" and "inspection frequency". Assuming a total of 5,200 concrete beam bridges are included in the bridge inspection program, 5,200 concrete beam bridges will be directly scheduled for inspection under the current bridge inspection scheduling method. In the selective bridge inspection scheduling method, 755 bridges are determined to be in poor technical condition and need to be inspected in time. The remaining 4,445 concrete beam bridges need to predict their deterioration state. If the inspection result is 0, the bridge will be inspected; otherwise, no inspection will be arranged. The predicted results are shown in Table 9.

**Table 9:** The prediction results of DT model.

Rating	Predicted level of deterioration	
	state-1	state-0
Deck system	3495	950
Superstructure	2782	1663
Substructure	1905	2540
Number of bridges to be inspected		2908

Data source: Calculated according to SPSS Modeler 18.1.

As can be seen from Table 9, of the 4445 bridges that have reached the regular inspection period, 2908 are in bad condition and the remaining 1537 are in good condition. That means 1,537 bridges will not be scheduled for regular inspection. As a result, instead of scheduling inspections for 5,200 concrete beam bridges, the number has been reduced to 3,663 (a reduction of 29.56%). This case study details the actual application of the deterioration state prediction model of concrete beam bridge in bridge inspection, which can provide an important reference for bridge maintenance personnel to use the deterioration state prediction model of concrete beam bridge. With the addition of screening mechanisms, the number of Bridges scheduled for regular inspection dropped dramatically. Bridges in poor condition are given higher priority and resources for inspection are allocated more scientifically.

### Establish a linkage inspection mechanism for neighboring assets

The deterioration of a traffic asset will reduce the deterioration of other traffic assets related to the traffic asset in terms of space or function [43]. Therefore, the study of the correlation between the deterioration state of adjacent traffic assets can provide an important reference for dealing with the linkage deterioration of adjacent assets caused by the interdependence of deterioration conditions.

#### (1) Dependency checks for adjacent assets

In this study, concrete beam bridges were taken as the main assets, while waterways, passageways and adjacent roads were taken as secondary assets. The correlation between each secondary asset and the main asset was tested successively, and the chi-square and Cramer’s V were selected as the test indicators. The null hypothesis H_0_ tested is that the conditional ratings of different assets are independent and have no interdependence with each other, and the significance level used is 0.01.

Chi-square and Cramer’s V tests were performed using IBM SPSS Statistics 26. As shown in Table 10, the P values of the chi-square test are all 0.000 and less than 0.01, rejecting the H_0_, indicating that there is a statistically significant dependence between the conditional ratings tested.

**Table 10:** Chi-square and Cramer’s V test results between adjacent asset conditions.

Input variables	Method	Concrete beam bridge		
		Deck system	Superstructure	Substructure
Waterway rating	Chi-square	.000	.000	.000
	Cramer’s V	.077	.095	.070
Passageway rating	Chi-square	.000	.000	.000
	Cramer’s V	.078	.097	**.432**
Adjacent road rating	Chi-square	.000	.000	.000
	Cramer’s V	.057	.054	.055

Data source: Calculated according to SPSS Statistics 26.

According to Cramer’s V’s tests, the dependence of concrete beam bridges on adjacent assets varies from 0.054 to 0.423. This means that a change in the conditions of a concrete beam bridge can cause a change in the adjacent assets to varying degrees, and vice versa. Among them, there is a strong interdependence between the substructure conditions and the passage conditions of the concrete beam bridge, which should be paid more attention.

#### (2) Linkage inspection mechanism for adjacent assets

Every traffic asset cannot exist independently, and the deterioration of the asset is not only caused by its own conditions or the direct action of the external environment, but also may be caused by the dysfunction or deterioration of the asset related to its function or space [43]. At present, the maintenance of different assets is independent of each other, and the coexistence and deterioration of assets is ignored. This maintenance management mode easily leads to the cost of a large number of resources to maintain the operating state of an asset, and its deterioration is the root of the deterioration of the conditions of its adjacent assets. Therefore, we need to look at the maintenance problem of concrete beam bridge from the perspective of connection. Firstly, the adjacent assets that are highly dependent on the concrete beam bridge are determined. When the bridge is inspected, the adjacent assets are inspected at the same time. The coexistence deterioration phenomenon is fully considered, and the deterioration condition of adjacent assets is included into the consideration range of the reasons for the deterioration of the bridge. For example, in Table 10, the substructure conditions of a concrete beam bridge have the strongest correlation with the passage conditions, and there may be coexisting deterioration among them.

**Table 11:** Example of concrete beam bridge having co-existing deteriorations.

Bridge code	Rating					
	Deck system	Superstructure	Substructure	Passageway	Waterway	Adjacent road
10600109705014	7	7	4	4	7	8
90740023202033	7	7	3	4	9	8
180710026002020	7	7	4	5	9	8
181750057402008	7	7	4	5	7	8
230470AA0392004	7	7	4	4	7	7
232150AA0146001	7	7	4	4	9	8

Data source: Screening of statistical data.

Of the 6 concrete beam bridges listed in Table 11, only the substructure and passageways are in need of maintenance, while other assets are in good condition. Due to the strong dependence between the substructure condition of the concrete beam bridge and the passageways condition, the possibility of coexisting deterioration phenomenon is greater. If only the lower structure of the concrete beam bridge is maintained and the passageways conditions remain unchanged, the state of the substructure will deteriorate rapidly due to the poor passage conditions, and vice versa. Therefore, the bridge management department should establish the sharing mechanism of inspection data between bridge and adjacent infrastructure. When predicting the deterioration of the bridge, the coexistence of deterioration should be fully considered, so as to grasp the state of the bridge more accurately and provide accurate information for the bridge maintenance.

## Conclusions

Bridge inspection is closely related to the sustainable development of bridges. However, the current bridge inspection mechanism in China is still not perfect in terms of science, accuracy and rational utilization of resources. Based on the prediction model of the deterioration state of concrete beam bridge, this paper puts forward suggestions on the optimization of the inspection mechanism of concrete beam bridge from three aspects of bridge inspection standard, inspection process and inspection content, and proves the feasibility of the suggestions through empirical analysis. It provides a new idea for the bridge management department to enhance bridge inspection efficiency and save bridge inspection resources. The main conclusions of this study are as follows:

1. DT has great potential in predicting future infrastructure conditions. This paper uses ANN, SVM and DT algorithms to predict the deterioration of concrete beam bridge. Accuracy, Recall, Precision and F-score were used to evaluate the prediction performance of the three algorithms. The results show that the prediction accuracy of ANN and DT is more than 90%, but DT has more advantages in computing speed. ANN has been widely used and proved to be effective by researchers in transportation assets condition predictions [15–17]. The exploration of DT in this paper can enrich the methodology of infrastructure deterioration state prediction.
2. It is suggested that the bridge management department should conduct multi-index differentiation inspection on the bridge according to the characteristics of the bridge and environment. It is found that bridge characteristics such as bridge age and structure length and environmental characteristics such as temperature, precipitation and wind speed have important effects on bridge deterioration prediction. And the concrete beam bridges with different degradation states have different statistical distributions in the above variables. This indicates that the above variables have the potential to become the standard for bridge inspection. Compared with the arrangement of bridge inspection plan only depending on the status of the bridge, it is more targeted to implement the multi-index differential bridge inspection. It is helpful to arrange the priority of bridge inspection reasonably and grasp the deterioration condition of various bridges in time. It is conducive to elaborating the inspection interval of various bridges and rationally allocating inspection resources.
3. It is suggested that the bridge management department should add a screening mechanism on the basis of the traditional bridge inspection process. The prediction model of concrete beam bridge deterioration condition is used to predict the bridge which is in good condition but reaches the inspection period in the regular inspection plan. Only those bridges with poor forecast results should be inspected. In the empirical analysis, the screening mechanism reduces the number of concrete beam bridges to be inspected from the original 5200 to 3663, saving nearly 30% of the bridge inspection resources. It is proved that this method can save inspection resources effectively. In addition, this study is optimized on the current bridge inspection process, which is easier to implement.
4. It is suggested that the bridge management department establish the linkage inspecting mechanism of adjacent assets to share the inspecting data of adjacent assets. The results show that there exists a co-existence deterioration between the substructure condition of the concrete beam bridge and the passageways condition, and the deterioration of the substructure condition of the concrete beam bridge will aggravate the passageways condition, and vice versa. The existing bridge management system has not yet conducted correlation analysis on the condition of the bridge and adjacent assets, but independently analyzed the deterioration state of each asset, and weighed the resource allocation problem on this basis. This paper fills this gap. The importance of establishing an adjacent asset linkage inspection mechanism is that it can reduce the chance of repeatedly maintaining the deteriorations of bridges that were actually caused by the deterioration of other assets.

In general, this paper has some inspiration for the bridge management department to improve the bridge inspection mechanism, improve the efficiency and effect of bridge inspection. Data collection in this study is limited. Further development will surely need to be supported by big data and advanced computer technology. There are still some deficiencies in this study, which can be further studied in the future: (1) Expansion and optimization of bridge deterioration prediction model; (2) Specific implementation measures for differentiated inspection of bridges; (3) In-depth exploration of the co-existence deterioration of bridges and adjacent assets.

## List of notation

ANN: Artificial Neural Network
CBR: Case-Based Reasoning
DT: Decision Tree
MLP: Multi-Layer Perceptron
NBI: National Bridge Inventory
RBF: Radial Basis Function
SVM: Support Vector Machine
UAV: Unmanned Aerial Vehicle
